# Entrepreneurship and innovation in clinical and translational science

**DOI:** 10.1017/cts.2021.881

**Published:** 2022-02-09

**Authors:** Robert Kimberly, Lars Berglund

**Affiliations:** 1 Department of Medicine, University of Alabama; 2 Department of Medicine, University of California, Davis

Innovation and out-of-the-box thinking are important drivers for clinical and translational science. The complexity of large health care systems and academic bureaucracy can make rapid and novel changes challenging. The often-used analogy of the challenges in quickly turning an ocean liner comes to mind. As the need to increase the speed for development of new diagnostic and therapeutic tools, to better implement such advances, to engage and reach the full spectrum of the population, and to train and educate the next generation is paramount, the question becomes how to best support these developments. One could argue that, in a broad sense, the launch of the CTSA initiative in 2005 was done to stimulate innovation in all of these areas on a national scale. Other NIH initiatives, such as the Centers for Accelerated Innovation (CAI) program at NHLBI represent large-scale top-down efforts in this direction. To ensure sustainable development and adoption of new concepts and technologies, academic health centers, often leveraging the CTSA initiative, have focused on building capacity locally including provision of tools such as training, project teams, and regulatory guidance as key factors. There are now vibrant bottom-up efforts at many such academic health centers to grow an academic culture that values entrepreneurship and innovation. The sharing of such experiences can help in accelerating successful approaches.

In the present JCTS thematic issue on Entrepreneurship and Innovation, a number of institutions and programs present their approaches and findings. The issue includes five reports from institutions on efforts at the local level (University of Colorado, University of Massachusetts Medical School in partnership with University of Massachusetts Lowell, University of Michigan, Case Western University, and Washington University). There are three reports from the NHLBI CAI program, two from sites (Boston Biomedical Innovation Center and the Cleveland Clinic) and one presenting evaluation of the CAI and the NIH Research Evaluation and Commercialization Hubs (REACH) programs. In addition, one paper describes the launch of the NCATS I-Corps program at 10 CTSA institutions and one paper from 4 CTSA centers detail efforts to develop a regulatory guidance program, the Regulatory Guidance for Academic Research of Drugs and Devices (REGARDD). Together, these papers illustrate the broad-based efforts throughout the nation to build capacity and to meet needs to reinvigorate the health care system. Undoubtedly, many more such efforts are ongoing at many places.

Some of the common threads in the papers are efforts to build expertise in product development and commercialization. As private industry has retreated to some extent from the early phases of translating discovery into marketable products, academic centers have started to fill this void. Many institutions are creating innovation centers where medical, engineering, and business faculty and trainees can come together and exchange ideas, as described in the reports from Massachusetts and Colorado. In many places, such an initiative also opens the door for company presence. Input from industry stakeholders is characteristic of several programs, such as the NHLBI CAI and I-Corps@NCATS programs. Success of such initiatives will require a culture of academic entrepreneurship – with an increased availability of well-trained faculty and investigators to navigate early phases of the multi-stage process of product development. Health systems and front-line clinic workers have much to add in assessing real-value propositions and the fit of proposed innovations to real-world needs. Many of the papers are focused on such capacity building – examples include the REGARDD program that presents efforts to reduce gaps in regulatory support, the I-Corps@NCATS program, modeled after the long-standing NSF program that encourages aspiring entrepreneurs to reach out to their potential customers, and the University of Michigan study that details efforts to implement training and funding mechanisms. An interesting feature of the latter was the involvement of patients and family members. Boots-on-the-ground experiential training and feedback seem more effective than didactic presentations alone. Funding, while critical, alone is insufficient, and both project-specific consultation and project development teams often play a critical catalytic role.

Working with existing university resources, such as Technology Transfer offices and Regulatory support programs, helps to accelerate efforts in these areas. The Boston report provides an example where the program was in part co-located with the institutional technology transfer office, facilitating expeditious interactions. The experiences detailed in these reports illustrate that while such existing offices and resources are important, there is a remaining need where CTSA and similar programs have important roles. In their role as an honest broker, institutional connector and change agent effector, these programs serve in a catalytic role that institutions can successfully leverage. Many of the papers in the issue illustrate such examples that bring us back to the original vision of CTSAs as engines of innovation and culture change. In developing and fostering a cadre of faculty with an entrepreneurial focus, there is also a need to critically review existing academic benchmarks for promotions and advancement. Contributions in the entrepreneurial area are likely to require benchmarks that go beyond publications, grants, and more traditional assessment tools. Such adjustments are under way at many places which is a welcome development.

We are pleased to present this thematic issue of JCTS which provides a synopsis of efforts to grow an institutional academic entrepreneurship culture. The examples illustrate the value of partnerships and multidisciplinary teams, and they underscore one of the important roles of the CTSA initiative. We encourage others to document and share their experiences as well.

